# Copper Nanowires as Highly Efficient and Recyclable Catalyst for Rapid Hydrogen Generation from Hydrolysis of Sodium Borohydride

**DOI:** 10.3390/nano10061153

**Published:** 2020-06-12

**Authors:** Aina Shasha Hashimi, Muhammad Amirul Nazhif Mohd Nohan, Siew Xian Chin, Poi Sim Khiew, Sarani Zakaria, Chin Hua Chia

**Affiliations:** 1 Materials Science Program, Department of Applied Physics, Faculty of Science and Technology, Universiti Kebangsaan Malaysia, Bangi 43600, Selangor, Malaysia; aina1shasha@gmail.com (A.S.H.); nazhifamirul@gmail.com (M.A.N.M.N.); szakaria@ukm.edu.my (S.Z.); 2 ASASIpintar Program, Pusat GENIUS@Pintar Negara, Universiti Kebangsaan Malaysia, Bangi 43600, Selangor, Malaysia; chinsiewxian@ukm.edu.my; 3 Center of Nanotechnology and Advanced Materials, Faculty of Engineering, University of Nottingham Malaysia Campus, Jalan Broga, Semenyih 43500, Selangor, Malaysia; PoiSim.Khiew@nottingham.edu.my

**Keywords:** acetic acid, catalytic activity, energy efficiency, H_2_ spillover, metal nanowires, NaBH_4_

## Abstract

Hydrogen (H_2_) is a clean energy carrier which can help to solve environmental issues with the depletion of fossil fuels. Sodium borohydride (NaBH_4_) is a promising candidate material for solid state hydrogen storage due to its huge hydrogen storage capacity and nontoxicity. However, the hydrolysis of NaBH_4_ usually requires expensive noble metal catalysts for a high H_2_ generation rate (HGR). Here, we synthesized high-aspect ratio copper nanowires (CuNWs) using a hydrothermal method and used them as the catalyst for the hydrolysis of NaBH_4_ to produce H_2_. The catalytic H_2_ generation demonstrated that 0.1 ng of CuNWs could achieve the highest volume of H_2_ gas in 240 min. The as-prepared CuNWs exhibited remarkable catalytic performance: the HGR of this study (2.7 × 10^10^ mL min^−1^ g^−1^) is ~3.27 × 10^7^ times higher than a previous study on a Cu-based catalyst. Furthermore, a low activation energy (E_a_) of 42.48 kJ mol^−1^ was calculated. Next, the retreated CuNWs showed an outstanding and stable performance for five consecutive cycles. Moreover, consistent catalytic activity was observed when the same CuNWs strip was used for four consecutive weeks. Based on the results obtained, we have shown that CuNWs can be a plausible candidate for the replacement of a costly catalyst for H_2_ generation.

## 1. Introduction

Lately, many researchers have been exploring the use of metal nanocrystals in applications such as catalysis, electrocatalysis, sensor design, antimicrobial materials, and flexible transparent electrodes [1,2,3,4,5,6,7]. Nanocoppers (Cu) of one-dimensional shape have received attention due to their high electrical conductivity [8]. Compared with noble metals, Cu is much cheaper and abundant [9], making it an attractive option to replace the highly expensive and scarce noble metals in various applications [10]. Due to their large surface area-to-volume ratio, Cu nanocrystals are progressively studied for catalysis applications such as the reduction of nitrophenols and H_2_ generation from the hydrolysis of NaBH_4_ [11,12].

The excessive consumption of fossil fuels has led to the deterioration of the ecological environment and a severe energy crisis, which in turn has increased the intensity of the search for safe, efficient, and clean energy sources. H_2_ is considered as a promising candidate to replace traditional fossil fuels due to its high energy density (142 MJ kg^−1^) and renewability, and its environmentally friendly by-product (water) [13,14]. H_2_ can be stored physically in liquid form or as a compressed gas. However, due to its low boiling and melting points, the pressure needed for the compression is too high which introduces the risk of leakage and explosion hazard [15]. Therefore, the lack of effectiveness, safety, and low cost of the H_2_ carrier limits its commercial availability worldwide [14].

On the other hand, chemically storing H_2_ in a solid state medium, such as a metal hydride, is potentially a safer and effective method [15,16]. There are several metal hydrides that have been utilized as chemical sources for H_2_ production, including sodium borohydride (NaBH_4_) [17] and lithium borohydride (LiBH_4_) [18]. NaBH_4_ is the most commonly utilized among all other metal hydrides because of its non-flammable and non-toxic nature [15]; it is also easy to be hydrolyzed [19,20], and its reaction product (NaBO_2_) is recyclable [21]. NaBH_4_ also exhibits high gravimetric/volumetric H_2_ storage capacity (10.8 wt%), which results in a high rate of H_2_ production [15,20,22]. One mol of NaBH_4_ can release 4 mol H_2_ gas: NaBH_4_ + 2 H_2_O → NaBO_2_ + 4 H_2_ (ΔH = −217 kJ mol^−1^) [21,23].

However, the hydrolysis of NaBH_4_ is slow at room temperature without a catalyst [24]. Therefore, various catalysts have been employed for the reaction [14,24,25,26,27]. Production of pure H_2_ can be obtained by the hydrolysis of NaBH_4_ with a controllable rate at ambient temperature with the presence of an appropriate catalyst. Previous studies showed that noble metals, such as platinum (Pt) [28], ruthenium (Ru) [29], and palladium (Pd) [30], are effective catalysts for the hydrolysis of NaBH_4_. Even though these catalysts show high stability and excellent catalytic activity, their expensive cost restrains their usage in wide applications [31]. The need to find durable, efficient, and cheap alternatives has led to further research works. Recently, there have been many studies done by using transition metal-based catalysts, such as cobalt [32], nickel [33], manganese [26], and Cu [12], to replace the noble metal catalysts. Some examples of transition metal-based catalysts are graphene-modified Co-B catalysts [14], Ni-Co-B hollow nanospheres [34], and cobalt boride@ nickel/reduced graphene oxide (Co-B@Ni/RGO) nanocomposites [35]. Even though there are many studies related to Cu alloys or composites [36,37,38], there is only one study reported on the utilization of a Cu-based catalyst for the hydrolysis of NaBH_4_ [12]. To the best of our knowledge, there has not been any study done as of this moment using CuNWs for the hydrolysis of NaBH_4_ to generate H_2_.

Even though there are advantages to using cheaper transition metals as catalysts, there are still several setbacks: these nanocatalysts are easy to aggregate which leads to a reduction in the specific surface area, thus causing a decrease in catalytic performance [32]. In addition, catalyst regeneration and leaching must also be addressed to increase the life span of the catalyst and availability for practical use. In this study, we synthesized copper nanowires (CuNWs) by a facile hydrothermal route, and we studied its catalytic performance in the hydrolysis of NaBH_4_ to produce H_2_ gas. Additionally, a short and simple treatment using glacial acetic acid (GAA) was conducted on the CuNWs. To avoid aggregation of the CuNWs during the catalytic reaction, the CuNWs were immobilized onto cotton cloth to improve the catalytic performance in terms of reusability, stability, and recoverability.

## 2. Materials and Methods

### 2.1. Materials

Copper chloride dihydrate (CuCl_2_, 2H_2_O, ≥99.0%), octadecylamine (ODA, C_18_H_39_N, ≥85.0%), sodium borohydride (NaBH_4_, ≥98%), and chloroform (CHCl_3_, ≥99.8%) were obtained from Merck. Ascorbic acid (AA, C_6_H_8_O_6_, ≥99%) was obtained from Sigma. Glacial acetic acid (GAA, CH_3_COOH, 99.85%) was obtained from HmbG Chemicals. Sodium hydroxide (NaOH, 99%) was obtained from SYSTERM. Cotton cloths (CC, 95% cotton) were cut into 0.5 × 1 cm^2^ for each hydrolysis reaction. All chemicals were used as received. All solutions were prepared with deionized water.

### 2.2. Synthesis of CuNWs

CuNWs were synthesized using a hydrothermal approach as reported previous [5]. Briefly, 26.3 mM of ODA, 2.8 mM of AA, and 5.6 mM of CuCl_2_·2H_2_O were dissolved in 30 mL of deionized water under stirring for 10 min and sonicated for 10 min. Next, the solution was transferred into a Teflon-lined autoclave and heated at 120 °C for 20 h. After the synthesis was done, the reddish-brown solution was washed with chloroform to separate CuNWs from other Cu products and kept in a sealed glass vial for further use.

### 2.3. Preparation of CuNWs Strips Samples

Different loadings of CuNWs (0.1 μg, 0.1 ng, 0.1 pg, and 0.1 fg) were drop-casted on cotton cloth strips (CuNWs/CC) and treated for 10 min using 10% GAA (GAA:isopropyl alcohol = 1:9) by dip coating. The CuNWs strips were kept in a sealed plastic bag and stored in a refrigerator.

### 2.4. Catalytic Study of H_2_ Generation

The kinetic studies of the hydrolysis of NaBH_4_ were carried out in a batch operation by the water displacement method, where the volume of H_2_ gas generated at a given interval is measured by reading the volume of the drained water in the cylindrical tube. In a typical measurement, 10 mL of 1 wt% NaBH_4_ that has been adjusted to pH 12 was prepared in a rubber sealed 50 mL conical flask. Then, a piece of CuNWs/CC with specific catalyst loading was put into the solution to initiate the catalytic reaction. The catalytic performance of the CuNWs/CC was tested by conducting several experiments at different pHs (10.45, 12, and 13), catalyst loadings (0.1 fg, 0.1 pg, 0.1 ng, and 0.1 μg), NaBH_4_ concentrations (0.1, 0.5, 1, 3, and 5 wt%), and reaction temperatures (298, 313, 323 and 333 K). Reusability and stability of the CuNWs/CC strips were investigated by repeating the GAA treatment using a freshly prepared NaBH_4_ solution. Continuous H_2_ generation by using a continuous flow system was also done by using 0.1 ng CuNWs with 10 mL of 1 wt% NaBH_4_. The continuous flow system was set up by using a syringe pump. NaBH_4_ was pumped at three different flow rates (2.5, 1.3, and 0.8 mL h^−1^) and passed through column reactor fitted on a HotCoil coil reactor (HotColumn™, Uniqsis, Cambridge, UK).

### 2.5. Characterization

The morphology of the CuNWs was analyzed by a field emission scanning electron microscope (FESEM, MERLIN ZEISS) and high-resolution transmission electron microscopy (HRTEM, FEI Technai G2 T20, Thermo Fisher, Waltham, MA, USA). The energy-dispersive X-ray (EDX) spectroscopy mapping was done by FESEM (FEI Quanta 400, Thermo Fisher). The chemical composition and crystal structure of the samples were examined by X-ray diffraction (XRD, Bruker D8 Advance) using Cu Kα radiation. The d-spacing (d) of the CuNWs was calculated using Bragg’s equation λ = 2d sin θ, where λ is the wavelength of X-ray radiation used, θ is the peak position angle, and d is the inter-planer distance.

## 3. Results

### 3.1. Characterization of CuNWs

The characterization of CuNWs using FESEM and XRD can be found from our previous study [5], where it can be seen that high-aspect ratio CuNWs (~2600) were successfully synthesized from the hydrothermal reaction. Figure S1a presents the HRTEM image of a nanowire that shows the lattice fringe spacing of 0.21 nm which corresponds to the d-spacing value of the (111) planes of fcc Cu [39,40] and is consistent with the previous XRD result. Figure S1b shows the EDX mapping of the Cu contents on the cotton cloth. It can be seen that there were only trace amounts of Cu added onto the cloth; due to the small size of the CuNWs, they could not be seen clearly among the fibers of the cotton cloth.

### 3.2. H_2_ Production

#### 3.2.1. Effects of pH

The hydrolysis of NaBH_4_ can be significantly influenced by the pH and temperature of the reaction. Compared with pure water, the hydrolysis of NaBH_4_ in water-alkaline solution is much slower due to the *in situ* simultaneous hydrolysis of sodium metaborate (NaBO_2_) which leads to the formation of NaOH, as shown in Equations (1) and (2) [41,42]:

NaBH_4(aq)_ + 2H_2_O_(l)_ → NaBO_2(aq)_ + 4H_2(g)_ (1)

NaBO_2(aq)_ + 2H_2_O_(l)_ → NaOH _(aq)_ + H_3_BO_3(aq)_ (2)

**Figure 1:**
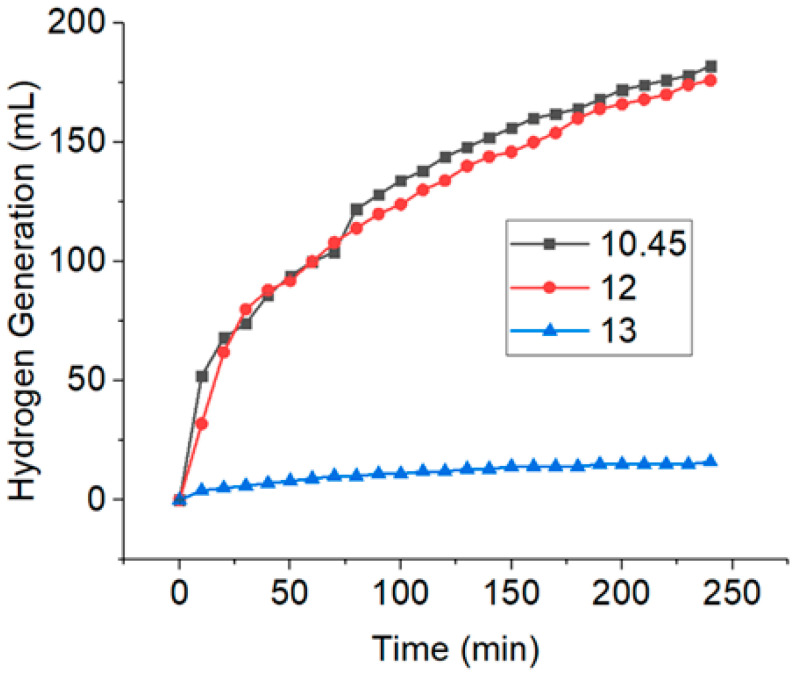
Plot of H_2_ generation versus time on the effect of different pH (conditions: 0.1 ng CuNWs, 10 mL of [NaBH_4_] = 1 wt%, and temperature = 298 K).

The importance of pH makes it the first parameter tested in this study. Figure 1 shows the H_2_ generation at three different pHs (10.45, 12, and 13). pH 10.45 was the initial value of the NaBH_4_ solution without any adjustments. NaOH (0.1 M) was added to obtain pH 12 and pH 13. The H_2_ generation at pH 10.45 and 12 was almost the same within the period of 240 min, meanwhile, at pH 13, the hydrolysis reaction was severely inhibited. This shows that H_2_ generation can be greatly suppressed with the addition of alkali substances [43]. Table 1 shows the list of HGRs obtained from the hydrolysis of NaBH_4_ at different pHs. It can be seen that the HGR value increased from pH 10.45 to 12, but decreased at pH 13. This is probably due to the involvement of OH^−^ in the hydrolysis of NaBH_4_. The catalyzed hydrolysis of NaBH_4_ can be accelerated with an appropriate increase in the NaOH concentration, thus enhancing the HGR. However, too much of NaOH could lead to a decrease in the solubility of NaBO_2_, thus causing the subsequent precipitation from the solution, adherence on the surface of the catalyst, and blockage of the active sites [33,44]. This would then hinder the contact of BH_4_^−^ with the catalyst surface, hence decreasing the hydrolysis rate [36]. With consideration of the real-life application in terms of the HGR, high H_2_ capacity, and long shelf life of the fuel solution, pH 12 was chosen as the optimized pH of the NaBH_4_ solution for further kinetics studies.

#### 3.2.2. Effects of Catalyst Loading

**Figure 2:**
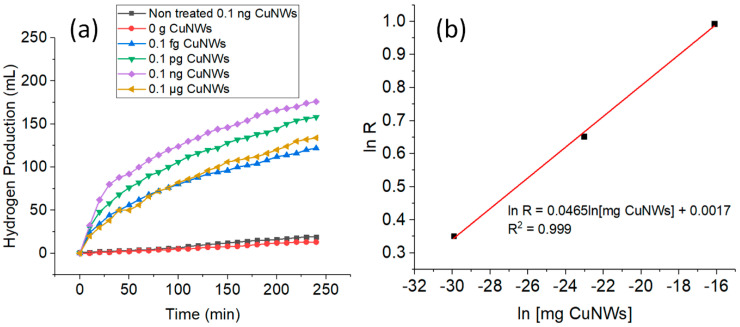
Plot of (**a**) different copper nanowires’ (CuNWs) loadings on H_2_ generation versus time; and (**b**) HGR versus CuNWs’ loadings, both in logarithmic scale. (Conditions: 10 mL of [NaBH_4_] = 1 wt%, pH = 12, and temperature = 298 K).

In the present study, CuNWs were investigated as a catalyst for H_2_ generation from an alkaline NaBH_4_ solution. Therefore, the effect of the CuNWs’ loadings was tested. Figure 2a shows the plot of H_2_ generation of different loadings of the catalyst with and without GAA treatment vs. time of reaction. It can be seen that there was very low H_2_ generation without GAA treatment on the CuNWs strips. This is probably due to the presence of residual ODA and a thin oxide layer on the surface of the CuNWs [9], which would prevent the direct contact of reactants with the active sites of the CuNWs. Even though the H_2_ generation using non-treated CuNWs strips was low, the total H_2_ generation in 240 min was slightly higher (19 mL) in comparison with when no catalyst was added to the solution (13 mL). After the treatment with GAA, the H_2_ generation increased significantly even with a very small amount of catalyst. This shows that the oxide and residual capping agent layers can be removed by using a short GAA treatment [45]. 

From Figure 2a, it can be seen that with the increase in catalyst loading (0–0.1 ng), the volume of H_2_ generation increases as well. When there was no catalyst added, the volume of H_2_ produced was only 13 mL in 240 min. According to the hydrolysis reactions, NaBH_4_ reacts slowly with water even without a catalyst to generate H_2_, even though it is not stable in air [43]. In comparison, when just a small amount of catalyst was added (0.1 fg), the volume of H_2_ generation increased significantly. In 240 min, the hydrolysis of NaBH_4_ for 0.1 fg, 0.1 pg, and 0.1 ng were 122, 158, and 176 mL, respectively. The enhancement in catalytic activity can be attributed to the increase in the specific surface area and more exposed active sites due to the addition of the CuNWs [17]. As the hydrolysis of NaBH_4_ proceeded, the rate in H_2_ generation started to decrease. During the hydrolysis process, the agglomeration of H_2_ and blockage of the active sites could impede the formation of new active sites for further catalytic cycles [17]. When the catalyst loading increased to 0.1 μg, only 134 mL of H_2_ gas was collected. This decrease in H_2_ generation is probably due to the agglomeration of excess CuNWs which could inhibit some of the active sites [46]. The rate of H_2_ generation was determined from the linear portion of each plot in Figure 2a. In this study, 0.1 ng was chosen as the fixed catalyst loading for the rest of the experiments due to easier handling and the volume of H_2_ generation obtained after 240 min was the highest. The HGR obtained for this study by using 0.1 ng CuNWs is 2.7 × 10^10^, and this value is ~3.27 × 10^7^ times higher than a previously reported Cu-based catalyst [12]. This indicates the importance of GAA treatment on CuNWs before the catalytic hydrolysis process. Figure 2b shows the values of the HGR versus the initial loadings of the CuNWs, both in logarithmic scale. The slope of the straight line is nearly zero (0.0465), indicating that the catalytic hydrolysis of NaBH_4_ is approximately zero order with respect to the catalyst loadings.

Presently, the dissociative chemisorption of BH_4_^−^ ions on the catalyst surface is generally accepted as the first kinetic step of the metal-catalyzed hydrolysis of NaBH_4_ [34,47]. Holbrook and Twist [48] suggested that the H_2_ was generated from both water and borohydride. Firstly, BH_4_^–^ ions are adsorbed on the electron-enriched Cu active sites of the CuNWs. The Cu-BH_4_^–^ ions then further dissociate to form Cu-BH_3_^–^ and Cu-H intermediates (Equation (3)). Subsequently, Cu-BH_3_^–^ reacts with H_2_O, possibly via the BH_3_ intermediate, to generate Cu-H and BH_3_(OH)^–^ (Equations (4)–(6)). After that, BH_3_(OH)^–^ undergoes a stepwise replacement of the B-H bonds by B-OH^−^, which then finally yields B(OH)_4_^–^. Next, the Cu-H species combines with another Cu-H to afford H_2_, and the active sites are regenerated (Equation (7)) [17,34].

(3) $${2\mathrm{Cu}+\mathrm{BH}}_{4}^{–}\;↔{{\;\mathrm{Cu}\text{-}\mathrm{BH}}_{3}}^{–}+\mathrm{Cu}\text{-}H$$

(4) $${\mathrm{Cu}\text{-}\mathrm{BH}}_{3}^{–}\;↔{\;\mathrm{BH}}_{3}{+\mathrm{Cu}+e}_{\mathrm{Cu}}^{–}$$

(5) $${\mathrm{BH}}_{3}{+\mathrm{OH}}^{–}\;\to {\;\mathrm{BH}}_{3}{\left(\mathrm{OH}\right)}^{–}$$

(6) $${\mathrm{Cu}+e}_{\mathrm{Cu}}^{–}{+H}_{2}O\;↔{\;\mathrm{Cu}\text{-}H+\mathrm{OH}}^{–}$$

(7) $$\mathrm{Cu}\text{-}H+\mathrm{Cu}\text{-}H\;\to {\;2\mathrm{Cu}+H}_{2}$$

#### 3.2.3. Effects of Concentrations of NaBH_4_

**Figure 3:**
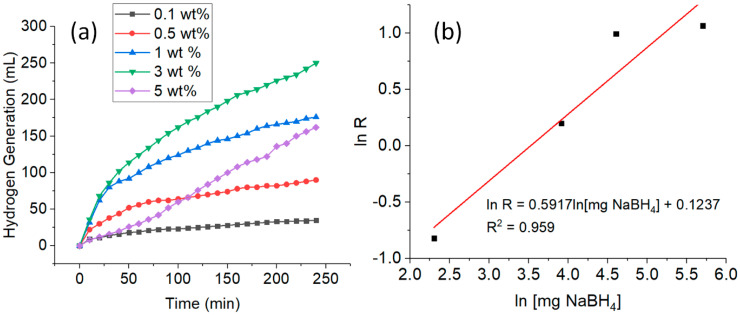
Plot of (**a**) different NaBH_4_ concentrations (wt%) on H_2_ generation versus time; and (**b**) HGR versus NaBH_4_ concentrations, both in logarithmic scale. (Conditions: CuNWs = 0.1 ng, pH = 12, and temperature = 298 K).

The effect of the initial NaBH_4_ concentration on the hydrolysis was studied by employing 0.1 ng CuNWs in an ambient condition. Figure 3a shows that the total H_2_ generation was 35, 90, 176, 250, and 162 mL by using 0.1, 0.5, 1, 3, and 5 wt% NaBH_4_, respectively. It can be seen that by increasing the NaBH_4_ concentration from 0.1 to 3 wt%, the H_2_ generation increased as well. However, at the NaBH_4_ concentration of 5 wt%, the volume of H_2_ generation decreased to 162 mL. This can also be seen with the HGR of the different concentrations of NaBH_4_ (Table 2), where the HGR increases with the increase in the NaBH_4_ concentrations (0.1–3 wt%), and then decreases when 5 wt% of NaBH_4_ is used. For the rest of the experiments, the NaBH_4_ concentration of 1 wt% was chosen for economic reasons.

Theoretically, a higher concentration of NaBH_4_ is desired to achieve a high capacity of H_2_. Therefore, when the NaBH_4_ concentration used is less than the highest HGR obtained, more H_2_O and BH_4_^–^ can be in contact with the active sites on the surface of the catalyst to generate H_2_ at higher NaBH_4_ concentrations. However, NaBO_2_ was produced simultaneously with H_2_. Due to the low solubility of NaBO_2_ under alkaline solution, NaBO_2_ accumulation on the surface of the catalyst and solution would occur at higher initial NaBH_4_ concentrations [46,49,50] Consequently, this will increase the solution viscosity [46,51] and further retard the mass transfer and decrease the H_2_ generation [33]. At higher concentrations of NaBH_4_, the insufficient active sites for the target reaction contributed to the lower catalytic performance [46]. 

Figure 3b shows the values of the HGR versus the initial concentration of NaBH_4_, both in logarithmic scale. The HGR in the catalytic hydrolysis of NaBH_4_ was calculated from the slope of each plot in the initial linear portion. Based on the slope of the straight line, it is indicating that the catalytic hydrolysis of NaBH_4_ is 0.59 order with respect to the concentrations of NaBH_4_, suggesting that the reaction follows fractional order kinetics with NaBH_4_ [42]. Consequently, the rate law of each catalyst for the catalytic hydrolysis of NaBH_4_ in this study can be given as in Equation (8):

(8) $$\frac{{-4d[\mathrm{NaBH}}_{4}]}{\mathrm{dt}}=\frac{{d[H}_{2}]}{\mathrm{dt}}{=k[\mathrm{CuNWs}]}^{0.05}{{[\mathrm{NaBH}}_{4}]}^{0.59}$$

#### 3.2.4. Effects of Temperature

**Figure 4:**
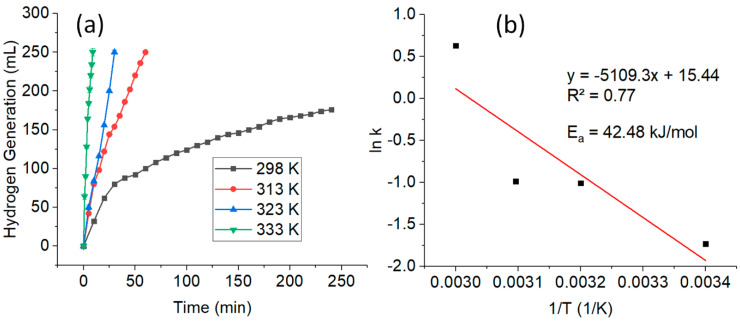
Plot of (**a**) different reaction temperatures on H_2_ generation versus time; and (**b**) the Arrhenius plot, ln k versus 1/T. (Conditions: CuNWs = 0.1 ng, 10 mL of [NaBH_4_] = 1 wt%, and pH = 12).

Considering the fact that the reaction temperature is an important factor influencing the hydrolysis kinetics of NaBH_4_, the effect of the reaction temperature was also investigated. Figure 4a shows the H_2_ generation by using CuNWs as the catalyst at a temperature ranging from 298 to 333 K. It can be observed that the H_2_ generation increases significantly with the reaction temperature. The time taken to reach a total volume of H_2_ generation of 250 mL was 60, 30, and 9 min at 313, 323, and 333 K, respectively. At an elevated temperature, more active reacting molecules are available which caused the faster H_2_ generation at a higher temperature [27]. 

The rate constants of H_2_ generation from the hydrolysis were measured from the linear portions of the H_2_ generation plots of the four different temperatures. These values were then used for the calculation of the activation energy (E_a_) from the Arrhenius plot. Figure 4b shows the plot of the log scale of rate constant and inverse temperature. The activation energy of NaBH_4_ hydrolysis catalyzed by CuNWs was determined from the Arrhenius equation (Equation (9)):

ln k = ln A − (E_a_/RT) (9)

where k is the reaction rate, A is the Arrhenius constant, E_a_ is the activation energy (kJ mol^−1^), R is the gas constant, and T is the absolute temperature (K). According to the slope of the straight line, the E_a_ obtained was 42.48 kJ mol^−1^. This value is comparable to the other values reported by using other non-noble metal-based catalysts (Table 3). The lower E_a_ of this study shows that a fast hydrolysis reaction rate was successfully achieved [14]. This indicates that CuNWs are a good and efficient catalyst for the hydrolysis of NaBH_4_.

#### 3.2.5. Reusability Test

**Figure 5:**
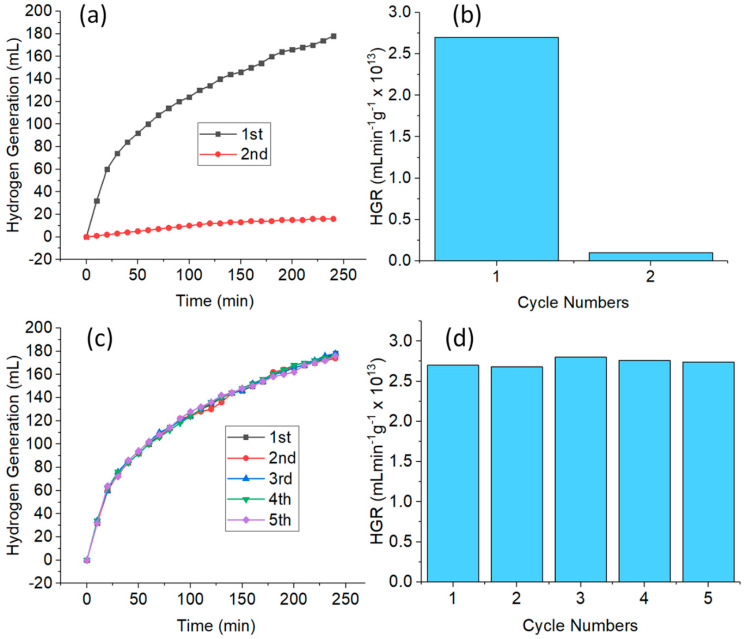
Plot of (**a**) H_2_ generation versus time; and (**b**) HGR of the hydrolysis of NaBH_4_ without retreatment of the CuNWs strips for two cycles, and (**c**) H_2_ generation versus time; and (**d**) HGR of NaBH_4_ with retreatment of the CuNWs using glacial acetic acid (GAA) in five consecutive cycles. (Conditions: CuNWs = 0.1 ng, 10 mL of [NaBH_4_] = 1 wt%, pH = 12, and temperature = 298 K).

The reusability of a catalyst is an important aspect to consider for stability, durability, and practical applications. Therefore, the reusability test of CuNWs as catalysts for the hydrolysis of NaBH_4_ was studied under similar experimental conditions. Figure 5a,b shows the H_2_ generation and HGR of the CuNWs without retreatment with GAA. By the second cycle, the catalytic performance of the CuNWs greatly decreased. In comparison with the first cycle where 176 mL of H_2_ gas was collected in 240 min, the total volume of H_2_ gas collected in the second cycle was only 16 mL. The volume of gas collected in the second cycle was only 9% of the initial cycle. This is probably due to the increase in boron products such as metaborate which hinder the accessibility of the active sites of the catalyst [36], thus decreasing its catalytic performance.

**Figure 6:**
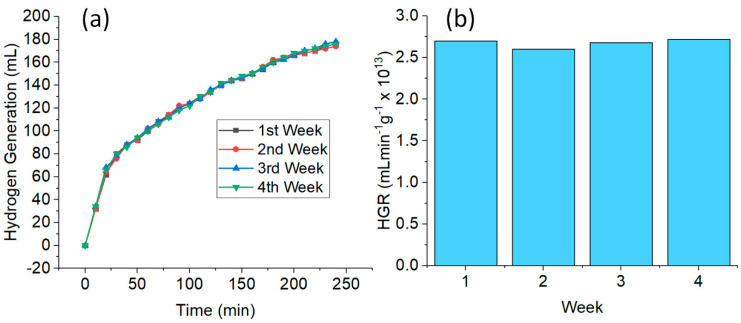
Plot of (**a**) H_2_ generation versus time; and (**b**) HGR of the hydrolysis of NaBH_4_ of the CuNWs strips for four weeks (conditions: CuNWs = 0.1 ng, 10 mL of [NaBH_4_] = 1 wt%, pH = 12, and temperature = 298 K).

Due to the lower catalytic performance of the CuNWs in the second cycle of the NaBH_4_ hydrolysis, the CuNWs strips were treated with GAA. Figure 5c,d shows the results of the reusability tests for the retreated CuNWs strips in five consecutive cycles. The volume of H_2_ gas generation and the HGR were stable and consistent throughout the entire cycles without any noticeable differences. This efficient catalytic performance can be attributed to the good retention of the CuNWs on the cotton cloth which enabled the good recoverability of the CuNWs. Figure 6a,b shows the plot of the same CuNWs strip used as a catalyst for every week in a month. The total volume of H_2_ generation and the HGR obtained in the span of a month were almost constant, which further shows the competence and stability of CuNWs as a catalyst in the hydrolysis of NaBH_4_.

#### 3.2.6. Catalytic Efficiency in Continuous Flow System

**Figure 7:**
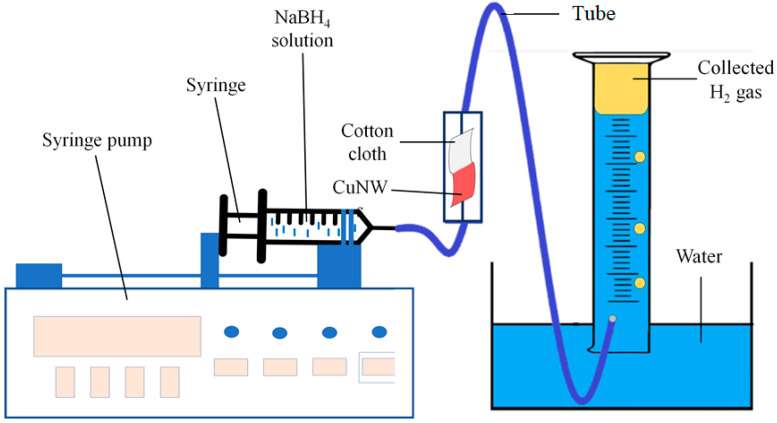
Schematic diagram of the continuous flow system setup for the production of H_2_ (conditions: CuNWs = 0.1 ng, [NaBH_4_] = 1 wt%, pH = 12, and temperature = 298 K).

The key advantage of a continuous flow system is the ability to accurately control the reaction parameters for a scale-up laboratory reaction [55,56]. Hence, it is significant to measure the catalytic efficiency of the continuous flow system proposed. The experimental setup is illustrated in Figure 7. Table 4 shows the total production of H_2_ obtained using the continuous flow system in the absence and presence of a catalyst at different flow rates. Based on Table 4, it can be seen that the H_2_ production increased significantly with the presence of the CuNWs. When the flow rate decreased, the total volume of H_2_ increased, which can be attributed to the increased retention time of the NaBH_4_ solution in contact with the CuNWs [57]. At the same time, the yield of H_2_ increased with the decrease in the flow rate applied. The results show that CuNWs can catalyze the hydrolysis of NaBH_4_ by using a continuous flow system. Table 5 lists the HGR for different production methods, showing the excellent catalytic performance of CuNWs in the production of H_2_ gas.

## 4. Conclusions

In this research work, a CuNWs catalyst was prepared by a simple hydrothermal method and used as a catalyst to produce H_2_ from the hydrolysis of NaBH_4_. An impressive catalytic performance was achieved by using a small amount (0.1 ng) of the catalyst, which yielded a high volume of H_2_ and the HGR was obtained. Furthermore, a low E_a_ (42.48 kJ mol^−1^) was calculated which shows the excellent catalytic activity of CuNWs. The effectiveness of the GAA treatment on the CuNWs before each reusability cycle was also shown. Consequently, the stable and consistent H_2_ generation and HGR for the reusability test up to five cycles and stability test for four weeks suggests that CuNWs are a suitable catalyst for practical applications. The continuous production of H_2_ could be a potential supply for proton-exchange membrane fuel cells.

## Figures and Tables

**Table 1:** List of H_2_ generation rate (HGR) values on different pH values.

pH	HGR (mL min^−1^ g^−1^ × 10^10^)
10.45	2.38
12	2.7
13	0.1

**Table 2:** List of HGR values of different NaBH_4_ concentrations.

Concentration of NaBH_4_ (wt%)	HGR (mLmin^−1^g^−1^ × 10^10^)
0.1	0.44
0.5	1.22
1	2.7
3	2.9
5	0.52

**Table 3:** List of comparisons of catalytic performance in this study with those reported in previous studies.

Catalyst	HGR (mL min^−1^ g^−1^)	E_a_ (kJ mol^−1^)	Reference
Cobalt/iron(II,III) oxide@carbon (Co/Fe_3_O_4_@C)	1403	49.2	[46]
Nickel-cobalt-boride (Ni–Co–B)	6400	33.1	[34]
Reduced graphene oxide-nickel (rGO-Ni)	33000	-	[52]
Silver/multi walled carbon nanotubes (Ag/MWCNT)	17.4	44.45	[53]
Palladium/multi walled carbon nanotubes (Pd/MWCNT)	23.0	62.66	[30]
Copper-ferum-boride (Cu-Fe-B)	-	57	[36]
Molybdenum disulfide/palm oil waste activated carbon (MoS_2_/POAC)	1170.66	39.1	[54]
Cobalt-cerium-boride/chitosan-derived carbon (Co-Ce-B/Chi-C)	4760	33.1	[24]
Cobalt-molybdenum/three dimensional graphene oxide (Co-Mo/3DGO)	7023.3	35.6	[27]
Nickel-cobalt-phosphorus/γ-aluminium oxide (Ni-Co-P/γ-Al_2_O_3_)	5180	52.05	[50]
Cu based catalyst	825	61.16	[12]
Copper oxide/cobalt(II, III) oxide (CuO/Co_3_O_4_)	6162.55	56.38	[37]
CuNWs (0.1 ng)	2.7 × 10^10^	42.48	This study

**Table 4:** Total volume and yield of H_2_ produced with different flow rates (with and without CuNWs).

Flow Rate (mL h^−1^)	Volume of H_2_ in the Absence of CuNWs (mL)	Volume of H_2_ in the Presence of CuNWs (mL)	Yield of H_2_ in the Presence of CuNWs (%)
2.5	9	49	18.85
1.3	19	158	60.77
0.8	26	238	91.54

**Table 5:** Comparison of HGR using different processes.

Method	HGR (mmol h^−1^)	References
Photo-fermentation	0.16	[58]
Direct bio-photolysis	0.07	[59]
Photocatalysis (visible light irradiation)	0.048	[60]
Photocatalytic decomposition of ammonia borane	0.022	[61]
Batch hydrolysis of NaBH_4_^1^	1.8	This work
Continuous hydrolysis of NaBH_4_ (flow rate = 0.8 mL h^−1^)	0.6	

^1^ Conditions: CuNWs = 0.1 ng, [NaBH_4_] = 1 wt%, pH = 12, and temperature = 298 K.

## Supplementary Materials

The following are available online at https://www.mdpi.com/2079-4991/10/6/1153/s1. Figure S1: HRTEM and EDX mapping of the CuNWs/CC sample.
