# Supplementary figures and images for: Copper Nanowires as Highly Efficient and Recyclable Catalyst for Rapid Hydrogen Generation from Hydrolysis of Sodium Borohydride
Source: Nanomaterials (Basel). 2020 Jun 12;10(6):1153. doi: 10.3390/nano10061153 (PMC7353277; doi:10.3390/nano10061153)

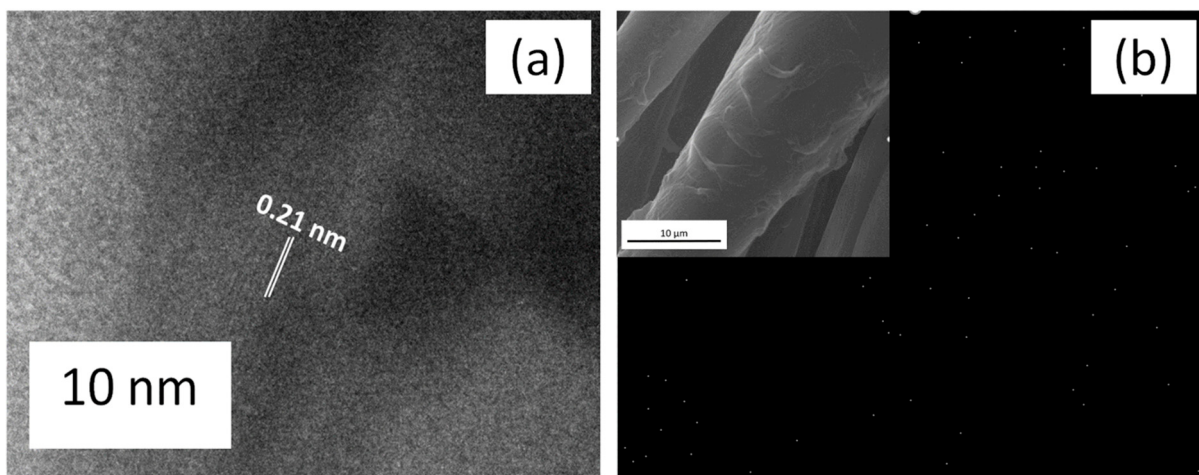

**Figure S1.** (a) HRTEM image of CuNWs, and (b) EDX mapping of the CuNWs/CC sample containing 0.1 pg CuNWs.

Supplement: Supplementary file 1 [file nanomaterials-10-01153-s001.pdf]
